# COMORBIDITIES ASSOCIATED WITH RISK OF MYOCARDITIS IN HOSPITALIZED COVID-19 OLDER ADULTS

**DOI:** 10.1093/geroni/igac059.3048

**Published:** 2022-12-20

**Authors:** Andrew Crawford, Andres Redondo, Ali Vaeli Zadeh, Alan Wong, Ricardo Criado Carrero, Alexander Fleischhacker, Elias Collado, Joshua Larned

**Affiliations:** Holy Cross Health-University of Miami, Fort Lauderdale, Florida, United States; University of Miami, Fort Lauderdale, Florida, United States; Holy Cross Health-Jim Moran Cardiovascular Research Institute, Fort Lauderdale, Florida, United States; Holy Cross Health-University of Miami, Fort Lauderdale, Florida, United States; University of Miami/Holy Cross Hospital, Fort Lauderdale, Florida, United States; University of Miami/Holy Cross Hospital, Fort Lauderdale, Florida, United States; Holy Cross Health-Jim Moran Cardiovascular Research Institute, Fort Lauderdale, Florida, United States; Holy Cross Health- University of Miami, Miami, Florida, United States

## Abstract

**Background:**

Older adults are afflicted more severely by COVID-19. SARS-CoV-2 can be complicated by myocarditis (MC), and the incidence of MC has been shown to correlate linearly with severity. However, data on comorbidities associated with MC in this population is scarce.

**Methods:**

Data were obtained from the PearlDiver database (PearlDiver Technologies, Fort Wayne, IN). The study used ICD codes to include patients hospitalized with a primary diagnosis of COVID-19, aged 65–75, and Elixhauser Comorbidity index(ECI)>4. Within this cohort, we identified patients diagnosed with MC 60 days after admission and compared their baseline comorbidities upon admission to those without MC. Pearson’s chi-squared test was used to compare groups. The strength of association was reported by Risk Ratios (RR). A p-value < 0.05 was deemed significant.

**Results:**

412,582 patients admitted with COVID-19 as the primary diagnosis were identified. 0.12% of this cohort developed MC over the following 60 days. The MC group was more likely to be male(57%, p=0.0001), with similar mean age(70.4, p=0.86) and mean ECI(9.4, p=0.07) to the no-MC group. Patients who developed MC have significantly higher rates of prior heart failure(RR= 1.30, CI95%=1.07–1.57, p=0.008). There was no difference between groups in terms of history of arrhythmias(p=0.36), cerebrovascular disease(p=0.09), chronic kidney disease(p=0.13), CAD(P=0.19), diabetes(p=0.48), ischemic heart disease(p=0.06), tobacco use(p=0.39), alcohol use(p=0.17), HIV(p=0.79), and severe liver disease(p=0.14).

**Conclusion:**

A history of heart failure increased the likelihood of developing MC in older adults.

